# Optimizing the medical equipment investment in primary care centres in rural China: evidence from a panel threshold model

**DOI:** 10.1186/s12913-024-10596-x

**Published:** 2024-02-01

**Authors:** Wanchun Xu, Zijing Pan, Liang Zhang, Shan Lu

**Affiliations:** 1 School of Medicine and Health Management, Tongji Medical College, Huazhong University of Science and Technology, No 13 Hangkong Road, Qiaokou District, 430030 Wuhan, Hubei China; 2 Sun Yat-sen University Cancer Centre, Guangzhou, China; 3 School of Political Science and Public Administration, Wuhan University, Wuhan, China; 4 Research Centre for Rural Health Service, Key Research Institute of Humanities & Social Sciences of Hubei Provincial Department of Education, Wuhan, China

**Keywords:** Equipment investment, Primary care, Resource allocation, Rural health care

## Abstract

**Background:**

The previous “one-size-fits-all” practice in resource allocation can no longer adapt to the spatial variation in population and health needs. This study aimed to investigate the spatially heterogeneous effect of medical equipment investment in the township health centres in rural China to optimize the investment strategies.

**Methods:**

Based on the national-scale stratified multistage cluster sampling, 319 township health centres from six provinces were included in the study. The retrospective data from 2013 to 2017 were collected for each sampled township health centres and the corresponding township community. The panel threshold regression model was applied to estimate the nonlinear effect of medical equipment increment on the service utilization due to the township communities’ urbanization degree. The influence of township community remoteness on the effects of equipment increment was investigated through subgroup analysis.

**Results:**

Among the township health centres in the neighbouring towns of the county seat (travel time to the county seat < 1 h), the significant effect of medical equipment increment was only found in the township health centres of the towns with high urbanization degrees (the proportion of the residents living in the built-up area > 69.89%), of which the effect size was 774.81 (95% CI 495.63, 1053.98, *p* < 0.05). Among the township health centres in the remote towns (travel time ≥ 1 h), the effect of medical equipment increment in the township health centres of the low urbanized towns (urban ≤ 5.99%, β = 1052.54, *p* < 0.01) was around four times the size of that of the counterparts (urban > 5.99%, β = 237.00, *p* < 0.01).

**Conclusion:**

This study demonstrated the spatially heterogeneous effect of medical equipment investment in the primary care centres in rural China. The priority of the equipment investment was suggested to be given to the township health centres in the remote towns with a low urbanization degree and those in the highly-urbanized neighbouring towns of the county seats.

## Background

Primary health care is an essential cornerstone of a sustainable health system [1, 2]. Strengthening the primary care system would contribute to the effectiveness of the health delivery systems and health equity among the population [3, 4]. The investment in equipment and other infrastructure is a fundamental measure in the capacity-building of primary care systems [5], which is also important for gaining the trust of residents and retaining local patients and staff in the primary care facilities in rural areas [6]. Enhancing and updating the medical equipment and other infrastructure of the primary care facilities is crucial toward the sustainable development of the primary care systems.

The Chinese government has greatly increased the investment in the rural primary care system in the new round of health reforms since 2009 [7]. Around 90,000,000 Chinese Yuan Renminbi (RMB) was invested in medical equipment and other infrastructure to strengthen the capacity of the primary care centres in rural China, which are also called township health centres (THCs) [8]. However, large variations existed in the effectiveness of the government investment in primary care facilities in rural China [9]. The decision-making process of the resource allocation is dominated by the health administrative department of the government, where the population size of the served areas has been the major consideration in the resource allocation for the THCs., The “Matthew Effect” was found to exist in the THCs in some regions due to the lack of evaluation of the actual service demand, which meant that the well-developed THCs would gain more investment due to their size and capacity, while the small THCs with weaker capacities would be in chronic underinvestment and thus in slow progress [9]. The medical equipment in some THCs was underutilized or even idle, while other township health centres still lack updated medical equipment [10, 11]. Similar situations were found in some middle-income countries, in which the budget allocation to the health sectors was primarily determined by the population size, rather than being guided by evidence-based or result-oriented approaches,, impairing the efficiency and equity of the health delivery systems [12]. Confronted with the increasing demand and the limited budget for health care, exploring strategies for optimizing the equipment investment on the primary care facilities in these developing areas is important.

Although abundant evidence has indicated the spatially heterogeneous effect of the government investment on the health system on large geographical scales in China [13, 14], few studies have investigated the potential factors that may influence the variance in the effectiveness and explored the optimization strategies. The utility of the resource input in the primary health care centres is mainly determined by the demand of the local areas [15]. However, the allocation of the medical equipment was generally related to the population size in the served areas and the qualification of the technicians of the THCs [16], which neglected the differences in the regional demand due to the variance in socio-economic development among various township communities. The identification of the key influencing factors on the township level from the spatial perspective, as well as the deeper understandings of their effect on the effectiveness of equipment investment, would contribute to the optimization of the allocation of medical resources. Remoteness and urbanization were the two most concerned spatial characteristics that may influence the effectiveness of equipment investment. The geographical location of the township communities has been gaining attention in regional health planning in rural China; [17] in which differential strategies were applied in the resource allocation for the THCs according to the degree of the remoteness of their served township communities in some pilot areas [18]. The urbanization in rural China also accelerated the differentiation of the township communities in terms of the socioeconomics and demographic characteristics, and the effect of urbanization on the volume and structure of the regional demand for primary care has also been discussed in some recent studies in China [19–21]. 

To our knowledge, the quantitative relationship between the spatial characteristics of the township community and the effectiveness of resource input in the THCs has not been evaluated yet. The relevant evidence could help in informing policymakers to develop specific differential strategies in the prospective resource allocation for primary care facilities. Most of the previous studies adopted the cluster method or subgroup analysis to investigate the heterogeneous effect of government investment on different types of THCs [13, 22], which may fail to fully show the quantitative relationship between the spatial characteristics and the effectiveness of the resource input. Therefore, the panel threshold regression model [23] would be used in the present study, which could help in estimating the threshold effect of the spatial variables on the relationship between equipment investment and service utilization in the THCs.

Based on the geographically representative data at the national level, the present study used the panel threshold model and urbanization degree of the township communities as a threshold variable to reveal the heterogeneous effect of the equipment investment on service utilization in the THCs. The influence of the remoteness of the township communities on the relationship between equipment investment and service utilization was also investigated in the study through the subgroup analysis. The findings of this study would help in informing the policymakers in China and other upper-middle-income countries (UMICs) with similar conditions to improve the investment policy and resource allocation for the primary care systems in the future.

## Methods

### Study design and sample

This study combined a retrospective data collection from the National Health Statistics Reporting System (2013–2017) and a cross-sectional survey (2018) for each participating THC based on a national-scale research project on primary care facilities in China. A combination of stratified multi-stage sampling and cluster sampling methods were applied in the survey. First, six provincial regions were selected based on their socio-economic level and geographical location (Eastern/Central/Western China). Second, one developed city and one underdeveloped city (based on the gross regional product per capita) were randomly selected in each provincial region. Third, one developed county and one underdeveloped county were randomly selected in each city. A total of 24 counties were sampled in the research project. All primary care facilities in the sampled counties/districts were investigated in the research projects, including 405 THCs from 24 counties.

Data of the sampled THCs from 2013 to 2017 were extracted from the National Health Statistics Reporting System, in which the health care facilities are required to submit a yearly report of their resources and services volume at the end of each year under the nationally unified statistical calibre. The demographic information of the sample township communities from 2013 to 2017 was extracted from the China Statistical Yearbook (Township). To ensure the accuracy and consistency in the linkage of these two data sources, complementary information was collected in a survey on the sampled THCs to confirm the accuracy of the linkage. The data extracted from the National Health Statistics Reporting System in one of the counties in Central China were missing due to regional data management policy, which was excluded from the analyses in the present study. Linear interpolation was used to fill in the missing value within the observations of each sampled unit in the panel data of this study. Cases (sample units) with missing values of the study variables every year were excluded from the analysis. The final sample size of the THCs in the present study was 319.

### Measures

#### Equipment investment

One of the most frequently used statistical indicators for the equipment investment in a medical facility or a region in China is the number of medical equipment that cost over 10,000 (RMB) (e.g. Digital Radiography X-ray system, Color Doppler ultrasound, Automatic biochemical analyzer) [24], which is also recorded in the aforementioned nationally unified report system. Therefore, the numbers of equipment each year in each sample THC were collected from the National Health Statistics Reporting System and used as the indicator to measure the equipment investment of the sample THCs. The effect of the annual increment (from 2013 to 2017) of this indicator will be estimated in the panel threshold model.

#### Service utilization of township health centres

Outpatient and emergency care is the major function of the primary care facilities in China, of which the improvement is widely considered to benefit the whole health system because it could help in reducing avoidable hospitalization and related medical costs in the general hospital [25]. Therefore, we used the annual volume of the outpatient and emergency visits as the dependent variable to measure the THC service utilization, of which the data were also extracted from the National Health Statistics Reporting System.

#### Urbanization degree of the township communities

The urbanization degree of each sampled township community was examined as the threshold variable in the present study. The urbanization in rural China has a peculiar pattern, which was driven by the industrialization in the rural areas (the development of the township enterprises) since the 1980s [26]. Therefore, the urbanization in rural China can be translated as “townification” (as opposed to the commonly used “citification”) [27], which can be defined as the concentration of the residents in the “built-up areas” of the township communities. Built-up areas refer to connected areas that have municipal and public facilities (e.g. industry, commercial, administration, and so on) and without agricultural activities, which include the following three areas: (a) The residential committee areas; (b) The village committee areas that are connected with public or residential facilities of the town; (c) Special areas such as independent industrial and mining areas, development zones, farms, and forest farms with a permanent population of over 3000 [28]. According to the literature review, the proportion of the residents living in the built-up area was used to indicate the degree of urbanization of the sampled township communities [29, 30]. To construct this indicator, we extracted the population size and the number of residents living in the “build-up area” of the sample towns from the China Statistical Yearbook (Township).

#### Remoteness of the township community

Given that the remoteness of the township communities is an important consideration in rural community health planning [17], the analysis was conducted in two separate subgroups. The travel time to the major cities is often used to classify the urban, peri-urban, and rural areas, of which the frequently used cut-off points comprise 30 min, one hour, three hours, and so on [31]. According to the township census by the Chinese National Bureau of Statistics, one of the indicators that measure the remoteness of a town is whether the travel time to the county government is less than one hour by the fastest local transportation method [32]. Therefore, we collected the travel time of each sampled township community to the county seat by bus in the cross-sectional survey and divided the THCs into two subgroups: (1) the THCs in the neighbouring towns of the county seat (travel time < 1 h); (2) the THCs in the remote towns (travel time ≥ 1 h). In China, the county seat and several township communities constitute a county.

#### Control variables

The control variables were selected from the potential confounding factors from the perspectives of the THCs and their corresponding township communities. The control variables were included as follows: (1) the population size of the served township communities; (2) the ageing degree of the township communities (the proportion of the population aged over 65); (3) the number of doctors in the THCs; and (4) the number of the medical technicians (including the laboratorians and medical imaging specialists) in the THCs. The population size of the served towns was collected from the China Statistical Yearbook (Township). Other control variables were extracted from the National Health Statistics Reporting System.

### Statistical analysis

A descriptive analysis was conducted to demonstrate the characteristics of the sampled township communities and THCs regarding the variables mentioned above. The THCs in the neighbouring towns of the county seat and the remote towns were analyzed separately.

We introduced the panel threshold regression model to investigate the nonlinear effect of equipment input on service utilization. Developed by Hansen, the panel threshold regression model has been widely used to identify the nonlinear effect of the investment or policies on the study objectives [23]. We used the model to test the threshold effect of the urbanization degree and search for the multiple regimes with regard to the various effects of the equipment input on the utilization of the THCs. The threshold effect of the urbanization degree was tested in the THC subgroups. The threshold value of the urbanization degree of each subgroup was obtained through the test, which would divide the THCs into the final subgroups. To obtain adequate power of the test regarding the regression results in the final subgroups, only the hypothesis of a single threshold was tested in the model. The *p*-value and 95% confidence interval (CI) of the threshold values were obtained through the bootstrap method. The significance level was set at 0.05 for the test of the threshold effect and the estimation of the coefficients in the regression model. The panel threshold model used in the present study was constructed as follows:

$$\begin{array}{l}{V}_{it}={\mu +\beta }_{11}{Input}_{it}\left(Urban\le \gamma \right)+{\beta }_{12}{Input}_{it}\left(Urban>\gamma \right)\\+{\beta }_{2}{Pop}_{it}+{\beta }_{3}{Aging}_{it}+{\beta }_{4}{Doc}_{it}+{\beta }_{5}{Tech}_{it}+{u}_{i}+{e}_{it}\end{array}$$

where,

$$ {V}_{it}$$: the annual volume of the outpatient and emergency visits in THC $$ i$$ in year *t*.

$$ {Input}_{it}$$: the number of medical equipment that cost over 10,000 (RMB) in THC $$ i$$ in year *t*.

$$ {Pop}_{it}$$: the population size of the served township community of THC $$ i$$ in year *t*.

$$ {Aging}_{it}$$: the ageing degree of the served township community of THC $$ i$$ in year *t* (the proportion of the population aged over 65).

$$ {Doc}_{it}$$: the number of the medical staff in THC $$ i$$ in year *t.*

$$ {Tech}_{it}$$: the number of medical technicians in THC $$ i$$ in year *t*.

*Urban*: the degree of urbanization of the served township communities (the proportion of the residents living in the built-up area).

$\gamma$: *the threshold value of the urbanization degree*.

## Results

### Characteristics of the sampled township communities and township health centres

The characteristics of the sampled township communities and THCs were summarized in Table 1. The neighbouring towns of the county seat had a larger population size. The population size had decreased by 2.11% in the neighbouring towns and up to 6.02% in the remote towns during the study period. The remote towns had experienced a greater pace of urbanization and ageing during the study period. The proportion of the population living in the built-up areas of the neighbouring towns had increased from 20.39 to 26.23%, which had risen from 17.30 to 26.90% in the remote towns.

**Table 1:** Characteristics of the sample township communities and township health centres (THCs) from 2013 to 2017

	THCs in neighboring towns (*n* = 175)			THCs in remote towns (*n* = 144)			
	2013	2017	Change	2013	2017	Change	
**Communities**							
Population size	36,187	35,422	-2.11%	22,727	21,359	-6.02%	
Ratio of population in built-up area (%)	20.39	26.23	28.64%	17.30	26.90	55.49%	
Ratio of population aged over 65 (%)	10.88	11.4	4.78%	10.79	12.35	14.46%	
**Township health centres**							
Number of medical equipment	13	18	38.46%	9	12	33.33%	
Number of doctors	20	22	10.00%	11	13	18.18%	
Number of medical technicians	2	3	50.00%	2	2	0.00%	
Annual volume of outpatient and emergency visits	36,258	38,710	6.76%	22,776	25,684	12.77%	

Notes: Values in Table 1 are presented as averages. THCs = township health centres

The average number of medical equipment (which costs over 10,000 RMB) in the THCs of the neighbouring towns had increased from 13 in 2013 to 18 in 2017, which increased by 38.46%. The average number of medical equipment was much smaller among the THCs in the remote towns (9 in 2013, 12 in 2017), and so was the number of doctors. Compared with the improvement in the equipment allocation, the increase in human resources was relatively slower in both THC subgroups, especially with the number of medical technicians (Table 1). The annual volume of outpatient and emergency visits was higher in the THCs in neighbouring towns of the county seat. During the study period, the service utilization of both THC subgroups had increased slightly (6.76% in the THCs of neighbouring towns vs. 12.77% in the THCs of remote towns).

### Test results of the threshold effects

Table 2 has shown the test results of the threshold effects of the urbanization degree in the relationship between the medical equipment input and service utilization in the THCs. The test was conducted in two THC subgroups separately. The threshold effect of urbanization was significant in both subgroups (*p* < 0.05), indicating that the utility of equipment input in the THCs varied due to the variance in the urbanization degree in the rural areas. Therefore, the panel threshold model seems to be more appropriate in the present study than the linear regression model. The threshold value of urbanization was 69.89% among the THCs in the neighbouring towns of the county seat (95% CI 68.87%, 70.87%), while the threshold value was 5.99% (95% CI 4.63%, 6.75%) in the subgroups of the THCs in the remote towns.

**Table 2:** Test results for threshold effect of urbanization in different township health centres (THCs) subgroups

Subgroup		F-value	*p*-value	Threshold estimates (%)	95% CI
THCs in the neighboring towns of the county seat		47.8	0.05	69.89	(68.87,70.87)
THCs in the remote towns		78.2	< 0.001	5.99	(4.63, 6.75)

Notes: A test for a threshold effect was conducted to determine whether the coeffecients of urbanization degree are the same in each regime. The null hypothesis and the alternatvie hypothesis (the linear versus the single-threshold model) are *H*_*0*_: *β*_*1*_ *= β*_*2*_; *H*_*a*_: *β*_*1*_ *≠ β*_*2*_. The F statistic is constructed as *F=(S*_*0*_*-S*_*1*_*)/ σ*^*2*^, where *S*_*1*_ and *S*_0_ represent the residual sum of squared errors with and without threshold effects, respectively, and *σ*^*2*^ represents the variance of the residual. We used bootstrap on the critical values of the F statistic to test the significance of the threshold effect (Pr = I(F > F1)). The method of bootstrapping with 300 samples were applied to obtain the confidential intervals of the threshold values with robust standard errors. THCs = township health centres

### Estimated effect sizes of medical equipment increment on the service utilization

Table 3 has shown the estimated effect size of equipment increment on the service utilization of the THCs at various regimes in each subgroup. Among the THCs in the neighbouring towns of the county seat, the significant effect of the medical equipment increment was only found in the THCs of the towns with high urbanization degree (the proportion of the residents living in the built-up area > 69.89%), of which the effect size was 774.81 (95% CI 495.63, 1053.98). However, the increment of the medical equipment had a universally positive effect on the THCs in the remote towns (*p* < 0.05), with the difference in the effect size in various regimes regarding the urbanization degree. Among the THCs in remote towns, the effect of the medical equipment increments in the THCs of the towns with low urbanization degree (regime 1: urban ≤ 5.99%, β = 1052.54, *p* < 0.01) was around four times the size of that of the counterparts (regime 2: urban > 5.99%, β = 237.00, *p* < 0.01).

**Table 3:** Estimates of the effects of the medical equipment input on the service utilization in different THC subgroups

	THCs in Neighboring towns of county seat (*N* = 175)			THCs in remote towns (*N* = 144)		
	Subgroup	β	95%CI	subgroup	β	95%CI
**Impact of input**						
Number of equipment	Regime1	155.23	(-66.41, 376.88)	Regime1	1052.54**	(830.30, 1,274.79)
	(urban ≤ 69.89%)			(urban ≤ 5.99%)		
	Regime2	774.81**	(495.63, 1,053.98)	Regime2	237.00**	(74.42, 399.59)
	(urban > 69.89%)			(urban > 5.99%)		
**Impact of covariates**						
Population size		-0.23	(-0.49, 0.03)		0.02	(-0.25, 0.29)
Aging degree		-320.05	(-772.28, 132.18)		-94.97	(-386.62, 196.68)
Number of doctors		112.39	(-124.15, 348.94)		114.09	(-89.59, 317.78)
Number of technicians		1316.73	(-52.29, 2,685.75)		520.52	(-380.40, 1,421.44)
**Intercept**		40776.59**	(28,990.76, 52,562.42)		19188.49**	(12,262.49, 26,114.49)
**Number of observation** ^**1**^		875			720	
**F-value** ^**2**^		8.96		16.09		
**Prob > F**		< 0.0001		< 0.0001		

Note: *p < 0.05 **p < 0.01. (1) Number of observations: the number of observations in the panel data, where the data for the variables were updated on a yearly basis; (2) F test indicates the overall significance of estimated regression model, where the null hypothesis is all of the regression coefficients are equal to zero.

## Discussion

### Discussion of the main results

According to the results of the present study, the effect size of the medical equipment increments on the service utilization of the THCs varied from place to place, depending on the geographical location and the urbanization degree of the township communities. There had been some empirical studies on the effectiveness of government investment in the health systems since the new round of Chinese health reform in 2009, which had exhibited the spatially heterogeneous effect of the government investment in the health systems on the provincial or regional (eastern/western/central China) scale [13, 14]. Using nationally representative data on the township level, the present study revealed the heterogeneous effect of equipment investment on the THCs in rural China. The findings indicated that the “one-size-fits-all” practice in resource allocation could not adapt to the spatial variation in health needs due to social development. Deeper understandings of the influencing factors of the utility of health resource investment are needed to optimize the prospective resource allocation, especially for the UMICs under rapid development.

The remoteness of the township communities is one of the major influencing factors of the utility of the equipment investment. Only a small amount of the THCs in the neighbouring towns of the county seat benefit from the equipment investment (only 7.43% of the THCs were in the high-urbanized regime of the neighbouring towns, according to the data on the urbanization degree of the sample towns in 2017). However, the significant effect of equipment investment was found in all subgroups of the THCs in remote towns, regardless of the urbanization degree. It was reported in the previous study that the technical efficiency of the THCs in rural China is positively related to the travel time to the general hospitals in the county seat [33], which indicated the greater cost-effectiveness of resource allocation in the THCs in remote towns. Some researchers found that the THCs in the neighbouring towns were more likely to be influenced by the “radiation effect” of the general hospitals or other medical institutions with higher quality in the county seat, which meant that the residents in the neighbouring towns would prefer to travel to the county seat to seek for better health care due to the relatively lower transportation costs, leading to the underutilization of the resource increment in these areas [34]. It is plausible that the improvement of the equipment in the THCs in remote rural areas is more likely to increase the service utilization of the residents. According to the data in this study, the THCs in the remote towns possessed less medical equipment than the THCs in the neighbouring towns during the study period, which indicated that the “diminishing marginal utility” may exist in the medical equipment investment [35]. The increment of medical equipment in the relatively poor-equipped THCs may have greater utility on the medical capacity and vice versa. The current status of the equipment should be taken into consideration in the prospective resource allocation for the THCs.

Another determinant of the utility of equipment investment is the urbanization degree of the township communities. The effect size of the equipment increment reached the highest in the THCs in the remote towns with lower urbanization degrees. It was also reported in the previous study that the scale efficiency of the THCs is negatively related to the proportion of the population living in the built-up areas [36], which indicated that the investment in the THCs in the less urbanized areas would have greater utility. The less urbanized towns were generally underdeveloped and may lack transportation, especially for those in remote areas. The less-urbanized remote towns also suffered from rapid ageing due to the emigration of young people. Despite the substantial demand for primary care, the THCs in these areas were usually suffering from chronic underinvestment due to the poor economy and fiscal capacity of the local government [37, 38]. Therefore, it can be inferred that the equipment investment in the THCs in such areas is more likely to improve the utilization of primary care by releasing the substantial unmet demand of the local residents. As for the neighbouring areas of the county seat, only the THCs in the towns with high urbanization degrees benefited from the medical equipment increment. The highly-urbanized towns near the county seat in rural China were usually highly industrialized, which would lead to the concentration of the population and improve their affordability and willingness to pay for health services [21, 39]. Compared with residents from neighbouring towns with lower urbanization degrees, most of the residents from highly-urbanized neighbouring towns lived in “built-up areas” and were closer to THCs, which would result in their higher accessibility to and utilization of health services from THCs. However, the primary care resources were found to be relatively inadequate in this urbanization process [40]. Similar situations also emerged in the built-up areas or the peri-urban areas in some middle-income countries, in which the health care resource was in relative scarcity due to the increase in healthcare demand [41, 42]. The positive effect of medical equipment investment in such areas suggested the importance of the capacity-building of the THCs in the highly urbanized towns in rural China, especially for the neighbouring town of the county seat.

### Implications for practice and research

The heterogeneous effects of the equipment investment in the THCs indicated that the “one-size-fits-all” investment policy could no longer adapt to the spatial variation in health needs due to social development. The findings of the present study could inform the policymakers of the following points to optimize the resource allocation for rural primary care centres. First, the priority of the medical equipment allocation should be given to the THCs in remote towns with low urbanization degrees. The utility of medical equipment investment reached the highest in such areas, and most of them were still suffering from underinvestment due to their economic status. Meanwhile, the competence of health personnel in utilizing the equipment also needs to be emphasized in rural THCs. Relevant policies such as training programs should be in place to facilitate the utilization of additional medical equipment. Second, the dynamic assessment of the demand for equipment should be emphasized to optimize the resource allocation for the THCs in the neighbouring towns of the county seat [33]. The effect of the medical equipment increment was insignificant in most THCs in the neighbouring towns of the county seat. The current status of the equipment of the THCs and the “radio effect” of the medical institution in the nearby county seat should be taken into consideration in the prospective resource allocation for these THCs. Third, attention should be paid to the capacity-building of the THCs in the highly urbanized neighbouring towns of the county seat. Similar to the situation in rural China, the development of the built-up areas and peri-urban areas in the countryside of some middle-income countries were also calling for the capacity of the local primary care systems to be improved to respond to the increasing health needs [41, 42]. 

The present study has significant implications for the research in the field of community-based health care planning in China and other UMICs. Deeper understandings of the influencing factors of the utility of health resource investment are needed to optimize the prospective resource allocation in these countries and regions. Although the methodology in the community-based health care planning for primary care has been developed in some high-income countries [43–45], the relevant evidence base and viable models in the context of the UMIC countries are still scarce due to the unavailability of the data and lack of studies, especially for the rural areas. The findings of this study could serve as a basis for future studies in this field.

### Limitations

The present study only explored how the geographical location and the urbanization degree of the township communities influenced the utility of medical equipment increment in the THCs. The other potential influencing factors, such as the population size of chronic disease patients, have not been included in this study, as reliable data could not be obtained for each sampled township community. However, we included the ageing degree, which may be positively related to the prevalence of chronic diseases as a control variable in the analysis. We may miss additional control variables (e.g. the economic status of the town) that are related to health demands due to a lack of data, which needs to be addressed in further studies. There may also be a bias in the measurement of the utilization level of the THCs by the residents because the minority of the residents in one township community may travel to another THC in the nearby township community to seek medical help. Further studies are needed to verify the findings of this study, which could use the real-world data extracted from the medical claims database to track the medical service utilization of the residents. Last but not least, it is important to acknowledge that the current findings of our study might be subject to the potential bias from the endogeneity. Future research endeavors are warranted to verify the results, including the identification and further data collection of suitable instrumental variables.

## Conclusion

This study demonstrated the spatially heterogeneous effect of equipment investment in primary care centres in rural China. The effect size of the medical equipment increment on the service utilization reached the highest in the THCs in the remote towns with lower urbanization degrees. The THCs in the remote towns with higher urbanization degrees also benefited from the equipment investment, but with a much smaller effect size. However, the effect of equipment increment was insignificant in most of the THCs in the neighbouring towns of the county seat. Only the THCs in the highly urbanized neighbouring towns of the county seat benefited from the equipment investment. The priority of the medical equipment allocation is suggested to be given to the THCs in the remote towns with low urbanization degrees and more attention should be paid to the capacity-building of the THCs in the highly-urbanized neighbouring towns of the county seat. Further research is needed to investigate the other potential spatial factors that might influence the utility of the resource input in the primary care facilities in the developing areas to optimize the prospective equipment investment.

## Data Availability

The datasets used and/or analysed during the current study are not publicly available due to the constraints on privacy and treatment imposed by the local regulatory authorities but are available from the corresponding author on reasonable request.

## Abbreviations

UMICs: Upper-middle-income countries
THCs: Township health centres
